# Detection of Mycotoxins in Patients with Chronic Fatigue Syndrome

**DOI:** 10.3390/toxins5040605

**Published:** 2013-04-11

**Authors:** Joseph H. Brewer, Jack D. Thrasher, David C. Straus, Roberta A. Madison, Dennis Hooper

**Affiliations:** 1 Plaza Infectious Disease and St. Luke’s Hospital, 4320 Wornall Road, Suite 440, Kansas City, MO 64111, USA; 2 Citrus Heights, CA 95610, USA; E-Mail: toxicologist1@msn.com; 3 Department of Immunology and Molecular Microbiology, Texas Tech University Health Sciences Center, Lubbock, TX 79430, USA; E-Mail: David.Straus@ttuhsc.edu; 4 California State University, Northridge, CA 91330, USA; E-Mail: vchsc001@csun.edu; 5 RealTime Laboratories, Carrollton, TX 75010, USA; E-Mail: dhooper@realtimelab.com

**Keywords:** mycotoxin, mold exposure, chronic fatigue syndrome, *Stachybotrys*

## Abstract

Over the past 20 years, exposure to mycotoxin producing mold has been recognized as a significant health risk. Scientific literature has demonstrated mycotoxins as possible causes of human disease in water-damaged buildings (WDB). This study was conducted to determine if selected mycotoxins could be identified in human urine from patients suffering from chronic fatigue syndrome (CFS). Patients (n = 112) with a prior diagnosis of CFS were evaluated for mold exposure and the presence of mycotoxins in their urine. Urine was tested for aflatoxins (AT), ochratoxin A (OTA) and macrocyclic trichothecenes (MT) using Enzyme Linked Immunosorbent Assays (ELISA). Urine specimens from 104 of 112 patients (93%) were positive for at least one mycotoxin (one in the equivocal range). Almost 30% of the cases had more than one mycotoxin present. OTA was the most prevalent mycotoxin detected (83%) with MT as the next most common (44%). Exposure histories indicated current and/or past exposure to WDB in over 90% of cases. Environmental testing was performed in the WDB from a subset of these patients. This testing revealed the presence of potentially mycotoxin producing mold species and mycotoxins in the environment of the WDB. Prior testing in a healthy control population with no history of exposure to a WDB or moldy environment (n = 55) by the same laboratory, utilizing the same methods, revealed no positive cases at the limits of detection.

## 1. Introduction

Chronic fatigue syndrome (CFS), also called myalgic encephalitis, has been widely studied over the past 25 years. Numerous mechanisms and theories have been proposed to explain its pathophysiology, epidemiology, clinical features and causation [1,2,3,4]. Possible causations include infections (particularly by viruses), oxidative stress, immune aberrations and toxic exposures, among others. However, no single etiology has been confirmed to fully explain this syndrome. In many circumstances, these patients remain chronically ill despite varying attempts at treatment [1,2,3,4].

During the same time frame, there has been a growing body of scientific literature indicating that mycotoxins and exposure to mycotoxin producing molds has become hazardous to the health of occupants of water-damaged buildings (WDB) (homes, schools and places of business). Water-damaged environments contain a complex mixture of biocontaminants produced by both mold, Gram-negative and Gram-positive bacteria [5]. Secondary metabolites of molds and bacteria have been identified in the dust, carpeting, wallpaper, heating, ventilation and air-conditioning (HVAC) systems and respirable airborne particulates [6,7,8,9,10,11,12,13,14,15,16]. In addition, mycotoxins have been identified in clinical isolates from corneal keratitis, aspergillosis and from body fluids and tissues of individuals exposed to moldy environments [17,18,19,20,21,22,23,24,25]. Interestingly, patients with mycotoxin exposure in WDB frequently have clinical features similar to CFS [5,26,27,28,29].

In this study, urine specimens were tested by ELISA-based assay to look for the presence of mycotoxins in a group of patients with CFS. These results were compared to healthy control subjects previously reported by the same testing laboratory. Additionally, in several cases, the WDB that were the source of exposure were investigated for environmental mold and/or mycotoxins. A hypothesis of possible mitochondrial damage in CFS is presented following review of the literature. 

## 2. Materials and Methods

### 2.1. Patients

The study was conducted for 6 months from 1 February 2012 to 31 July 2012. Patients with chronic illnesses, many of whom were previously diagnosed with CFS, were seen in a private practice (JHB) which is a consultative outpatient infectious disease clinic in Kansas City, Missouri. Out of approximately 300 patients with chronic illness that were seen for routine follow up clinic visit, 112 met the criteria for a diagnosis of CFS as outlined by Fikuda, *et al.* in 1994 [4]. These patients were from diverse geographic areas in the United States however, the majority resided in Midwestern states. The patient ages ranged from 15 to 72 years with 84 (75%) females and 38 (25%) males. The duration of symptoms ranged from 2 to 36 years with an average duration of 7.8 years. The illness was so severe that 76 (68%) of the patients were either unable to work, receiving disability or unable to attend school. A past history of mold allergy and/or chronic sinusitis was present in 50 (48%) of the patients. These patients failed to respond to treatments and their CFS symptoms lingered. 

Common symptoms in this patient population included fatigue, headache, flu-like symptoms, cognitive complaints, myalgia, arthralgia, gastrointestinal problems and various neurologic symptoms. Other previous diagnoses included fibromyalgia, Lyme disease, peripheral neuropathy, orthostatic intolerance (including postural orthostatic tachycardia syndrome and neural-mediated hypotension), migraine, chronic dermatitis, gastroparesis, chronic abdominal pain, irritable bowel syndrome, interstitial cystitis, anxiety, depression, chemical sensitivity, vertigo, chronic sinusitis, gluten intolerance, tremor, myoclonus and cognitive dysfunction.

Routine laboratory parameters including complete blood count and chemistry panels were usually normal. Immune testing had been performed previously in most of these patients. The most common abnormality was diminished natural killer cell (NK) function. Other immune abnormalities were occasionally noted (e.g., hypogammaglobulinemia).

Since these chronic conditions have been reported to be associated with exposure to mold and bacteria in WDB and previous studies have shown an association between CFS and sick building syndrome (SBS), it was decided to carry out an environmental history and discuss urine mycotoxin testing [28]. During these follow up visits, over 90% of the 112 patients confirmed exposure to a WDB and frequently the presence of a moldy environments in the home, workplace or both.

### 2.2. Control Subjects

Healthy control patients with no known toxic mold exposures in water-damaged buildings were previously reported [21]. These controls (n = 55) consisted of 28 males and 27 females, ages 18 to 72 years. These patients were also from diverse geographic areas and resided in various areas of the United States. Urine specimens from these individuals were used to develop reference data for the control group used in this study. Furthermore, the same control subjects were also asked about complaints and/or symptoms related to mold exposure as documented in the peer reviewed literature at the time of this study [30]. Symptoms that were screened included rhinitis, cough, headache, respiratory symptoms, central nervous system symptoms, and fatigue. They did not give a history of water-intrusion or mold growth in the workplace or at home. It was assumed that the controls had exposure to foods and airborne mold spores that occur in their daily activity.

### 2.3. Mycotoxin Testing

Mycotoxin determination was conducted in similar fashion as described earlier with modifications [21]. Competitive direct enzyme linked immunosorbant assays (ELISAs) were conducted on all groups of mycotoxins studied (AT, OTA, MT). Validated, competitive direct ELISA tests for MT and AT/OTA (private communication, RealTime Laboratories, Inc, Carrollton, TX) were conducted on all urines submitted [21]. Validations have demonstrated that urine is the best fluid for evaluation. However, the variability of urine matrix components such as organic compounds, pH and electrolytes can affect antibody binding and assay performance in ELISA tests. To account for these matrix effects, standard sample diluents for plasma serum, cell culture and other biological specimens have been developed. No standard diluent has been developed for urine and many other biological fluids. Instead, phosphate buffered saline (PBS) was used as a “urine-like” diluent. In the validations and continued testing of mycotoxins, a 10% methanol in PBS +10% methanol dilution (pH 7.2–7.4) was used to compensate for the matrix effect in urine. 

MT ELISA test: Urines were diluted at 1:5 to compensate for the matrix affect. Coated roridin A (an MT) antibody ELISA wells (Beacon Analytic Inc., Maine) were inoculated with 100 microliters (µL) of controls, calibrators, or patients specimens. One hundred microliters of diluted urine were placed in each test well. One hundred µL of known MT (roridin A, Sigma Inc., St. Louis, MO) calibrators (diluted: 10.0 µL/dL, 1.0 µL/dL, and 0.1 µL/dL, 0 µL/dL) and high, low, and negative controls were placed in specific wells. Samples were incubated for 15 minutes at 21–25 degrees C under continual rotation. One hundred µL of 1:1800 dilution of roridin A HRP-conjugate (Beacon Analytic Inc., Maine) were added to each well and incubated for 15 minutes at 21–25 degrees C, under continual mild rotation. All plates were washed 4 times with deionized water and tapped until deionized water was removed. One hundred µL of substrate (Beacon Analytical Labs, Inc.) were placed in each well and incubated 30 minutes at 21–25 degrees C under continual rotation. One hundred µL of 1 N HCl were added to stop the reaction. The reactions were read on a Spectra Max 190 Spectrophotometer (Molecular Devices, Sunnyvale, CA) at 450 nm. Results were tabulated and entered into a semilog software program (Beacon Analytical Labs, Inc.). Results were tabulated and reported as ng/dL or parts per billion (ppb). All controls and calibrators met regulatory conditions as specified in the standard operating procedures. 

AT and OTA ELISA procedures: Urines were diluted at 1:7 to compensate for the matrix affect. Coated wells (Neogen Corporation, Michigan) with either polyclonal antibodies to AT or OTA were used in the separate mycotoxin procedures. Procedures for AT and OTA determinations were identical except for the specific antibody coating the ELISA plates. Antibody ELISA wells (Neogen Corporation) were inoculated with 100 µL of calibrators, controls or patients specimens. Initially, 100 µL of AT-HS conjugate (Neogen) and 100 µL of OTA conjugate (Neogen) were placed in the respective antibody wells. One hundred µL of known antigen (AT or OTA, Trilogy Inc, MO) calibrators for AT were 0, 1, 2, 4, and 8 ng/dL (ppb) and calibrators for OTA were 0, 2, 5, 10, 25 ng/dL (ppb). High, low, and negative controls for each mycotoxin were also placed in specific wells. One hundred µL of diluted urine were placed in each test well. Plates were incubated at 21–25 degrees C for 10 minutes under continual mild rotation. All plates were washed 4 times with deionized water and tapped until deionized water was removed. One hundred µL of substrate (Neogen) were placed in each well and incubated 10 minutes at 21–25 degrees C under continual mild rotation. One hundred 100 µL of 1N H_2_SO_4_ were added to stop the reaction. The reactions were read on a Spectra Max 190 Spectrophotometer (Molecular Devices, Sunnyvale, CA) at 650 nm. Results were tabulated and entered into a semilog software program (Neogen Inc). Results were tabulated and reported as ng/dL or ppb. All controls and calibrators met regulatory conditions as specified in the standard operating procedures.

### 2.4. Statistics

Statistics were performed on the patient data and controls for each of the three mycotoxins (AT, OTA and MT). Two-sided independent t-tests were performed on OTA and the MT. Two-sided Fischer exact test was performed on the AT because the control data were negative (zero) for this group.

## 3. Results and Discussion

Mycotoxin testing revealed the presence of at least one of the toxins in the urine of 104 out of 112 (93%) patients. This included 103 with positive results and one that was in the equivocal range for OTA. The frequency of the various mycotoxins in the urine of CFS patients (based upon the suggested detection limits of RealTime Laboratories) is summarized Table 1. OTA was most commonly detected mycotoxin comprising 83% of the patients. This was followed by MT (44%) and AT (12%). The presence of combinations of mycotoxins in the urine were follows: OTA + MT (23%), AT + MT (4%), and all three (8%).

**Table 1:** This table summarizes the detection of the mycotoxins in the urine of chronic fatigue syndrome (CFS) patients individually or in combinations. The ranges and averages are based upon the actual number of individual positives for each mycotoxin.

Mycotoxin	Positive (N, %)	Range (ppb)	Average (ppb)
AT^a^	13, 12%	1.1–9.4	4.67
OTA^a^	87, 83%	2–14.6	6.2
MT^a^	46, 44%	0.21–5.72	0.85
OTA + MT	24, 23%	N/A^b^	N/A^b^
AT + MT	4, 4%	N/A^b^	N/A^b^
AT, OTA, MT	8, 8%	N/A^b^	N/A^b^

a: Limits of Detection: AT (1 ppb); OTA (2.0 ppb); MT (0.2 ppb). b: N/A: Not applicable.

The CFS patients were compared to a previously published group of healthy control subjects that had no history of exposure to a WDB or moldy environment. The frequency of detection of these three mycotoxins in the CFS patients compared to controls is seen in Table 2. 

**Table 2:** Detection of mycotoxins in CFS patients compared to healthy controls.

Patient Group	Number Tested	AT^a,b^	OTA^a,b^	MT^a,b^	Any Mycotoxin^b^
CFS	112	12 (12%)	87 (83%)	46 (44%)	104 (93%)
Control^c^	55	0	0	0	0

a: Limits of Detection: same as Table 1. b: Number positive, percent positive; c: Control group previously published [21].

The concentration of mycotoxins in the urine of patients and controls were statistically analyzed to determine if a difference existed between the two groups. These data are summarized in Table 3. The concentrations were significantly elevated in the patients compared to controls as follows: AT (0.43 ± 1.36 *vs.* 0 ± 0 ppb, *p* = 0.0007), OTA (5.26 ± 3.65 *vs.* 0.355 ± 0.457 ppb, *p* < 0.0001), and MT (0.422 ± 0.714 *vs.* 0.0169 ± 0.0265 ppb, *p* < 0.001).

**Table 3:** This table summarizes the independent two tailed t-tests performed on the patients and controls with respect to ochratoxin A (OTA) and macrocyclic trichothecenes (MT). The Fisher 2-Sided Exact test was performed on the aflatoxins (AT) because the control group had non-detection of AT. The mean and standard deviations are listed in ppb for each mycotoxin (patients and controls).

Mycotoxin	Patients (N = 104) (ppb)	Controls (N = 55) (ppb)	*t*-value	p
AT	0.43 ± 1.36	0 ± 0	-----	0.0007^a^
OTA	5.26 ± 3.65	0.355 ± 0.457	13.5	<0.0001
MT	0.422 ± 0.714	0.0169 ± 0.0265	5.78	<0.001

a: Fisher 2-Sided Exact Test Matrix: Controls (55 and 0); Patients (87 and 17).

Environmental histories of these patients were positive for exposure to WDB (many with visible mold) in over 90% of the cases tested, including residential and/or workplace. In the residential group, water damage to the basement was a common finding. However, other sources of water intrusion were noted during history taking, which included water pipe leaks, roof leaks, window leaks and plugged drains. In 24 patients, symptoms, which eventually became chronic, started within one year of the exposure in the WDB.

Environmental tests (air spore counts, tape lifts and the examination of dust for mycotoxins) were performed in 10 of the situations of the 104 patients (data not shown). In addition, two families discussed below also conducted environmental testing. In the 10 cases mold genera associated with the potential for mycotoxin production were found. In 8 of the situations, *Stachybotrys* was identified in the WDB. In each of these 8 patients, MT was detected in the urine assay. In addition, *Aspergillus/Penicillium*-like spores were detected in 8 buildings to which these patients were exposed. The urine mycotoxin assays identified OTA in 5 patients and AT was present in 2 subjects. Additionally, dust specimens collected from 5 homes and one office building were sent to RealTime Laboratories for mycotoxin testing on environmental dust. MT was found in the dust samples from all 6 of these buildings. Small amounts of OTA were detected in 4 of the dust samples. There were 7 patients that had been exposed to mold in these buildings. Of these 7 patients, 6 had tested positive for MT in the urine assay, with the levels ranging from 0.21 ppb to 5.72 ppb. Additionally, 4 of the 7 patients had tested positive for OTA with values ranging from 3.7 ppb to 10.2 ppb. 

The two families that conducted environmental tests on their homes are presented below. The families consisted of four individuals per household (Table 4, Table 5). 

Family #1**: **The parents moved into a new home in 1991 and the family has lived there since. The father began to develop symptoms of fatigue, muscle aches and cognitive problems, which was subsequently diagnosed as CFS, within 4 months of moving into their new home. Within 3 years, the mother developed CFS. Neither of the parents had any history to suggest occupational exposure outside of the home. The two daughters were born and raised in that home. Both children developed chronic illness (CFS) while living in the home. All four remain chronically ill (CFS). Urine AT, OTA and MT concentrations (ppb) for each family member were as follows: father 0, 0, 0.59; mother 0, 3.6, 0.19; daughter 0, 4.2, 0.13 and another daughter 0, 3.6 and 0.17. Table 4 summarizes the results from this family.

**Table 4:** Detection of various mycotoxins in the urine of members of Family #1.

Family member	Age	Sex	AT^a^ (ppb)	OTA^a^ (ppb)	MT^a^ (ppb)
Father	50	M	0	0	0.59
Mother	49	F	0	3.6	0.19
Child	19	F	0	4.2	0.13
Child	16	F	0	3.6	0.17

a: Limits of detection same as Table 1.

Standard air testing from their home revealed high numbers of *Aspergillus* spores in one area of the home. Dust samples were collected on three different occasions from top of doorway jambs, kitchen cabinets, bedroom, living room, kitchen, and home office and sent to Mycometrics LLC, (Monmouth Junction NJ) for MSQPCR-36 (ERMI) testing [31]. The moldiness indices for these samples were 8.61, 16.54 and 16.7. Mycotoxin producing molds identified in dust samples were as follows: *Aspergillus* (*flavus, fumigatus, niger, ochraceus* and *versicolor*)*; Penicillium* (*brevicompactum, purpurogenum, crustosum, corylophilum* and *chrysogenum*)*; Chaetomium globosum, Stachybotrys chartarum* and *Trichoderma viride*. A dust sample from under the refrigerator sent to RealTime Laboratories for mycotoxin testing revealed MT (0.42 ppb) and OTA (0.6 ppb). The family had no idea there was a “mold problem” in their home until 2012 when the environmental testing was completed.

Family #2**: **All members of this family had chronic illness (CFS, celiac disease, chemical hypersensitivity) which had developed after living in this home. The family moved into a home in 1997 and within months discovered problems with the exterior drainage which led to water intrusion. There was subsequent flooding of the lower level of the home on multiple occasions. Environmental air sampling of the home in 2005 revealed *Aspergillus*/*Penicillium*-like spores and *Stachybotrys*-spores. The family moved to a different home in 2005 (within months of the testing results) but all remained ill. The father had no history of occupational exposure outside the home and the mother did not work outside the home during this time frame. The urine mycotoxin levels (ppb) for AT, OTA, and MT in this family were as follows: father 0, 4.6, 0.02; mother 0, 6.8, 0.01; son 0.5, 6.1, 0.48 and daughter 0, 2.3, 0.03. The results for this family are seen in Table 5.

**Table 5:** Detection of various mycotoxins in the urine of members of Family #2.

Family member	Age	Sex	AT^a^ (ppb)	OTA^a^ (ppb)	MT^a^ (ppb)
Father	49	M	0	4.6	0.02
Mother	54	F	0	6.8	0.01
Child	23	M	0.5	6.1	0.48
Child	15	F	0	2.3	0.03

a: Limits of detection same as Table 1.

The etiology of CFS has been studied for several decades and numerous proposed etiologies have been suggested [1,2,3,4]. Studies of CFS patients have demonstrated evidence of increased viral activation, oxidative stress, immune abnormalities, neurocognitive features and endocrine abnormalities [1,2]. In addition, CFS patients have mitochondrial dysfunction with impaired oxidative phosphorylation, low ATP stores and increased lactic acid with exercise [32,33,34]. Individuals that have been exposed to WDB frequently have clinical features similar to CFS [5,22,23,24,25,26,27,28,29]. One study reported concurrent sick building syndrome and CFS [28]. However, it should be recognized that additional symptoms in CFS patients include fibromyalgia, headaches, loss of balance, neurocognitive difficulties, flu-like symptoms, irritable bowel syndrome, anxiety, depression, among others symptoms. 

In this study, patients with a prior diagnosis of CFS were evaluated for the presence of mycotoxins utilizing a sensitive and specific ELISA-based urine assay for three common mycotoxins. Ninety-three percent of the cases demonstrated the presence of at least one of the mycotoxins in the urine. Over 90% of the patients gave a history of exposure to WDB. Additionally, mycotoxin-producing mold species, mycotoxins or both were demonstrated in WDB that were associated with exposures in 18 of these patients. The demonstration of the actual toxins in dust samples from the buildings in which the patients either lived or worked is of considerable interest. This is because the same mycotoxins were recovered from the urine of these patients. Trichothecene mycotoxins can be found in small fragments as well as in conidia [7]. Furthermore, a variety of mycotoxins and bacterial exotoxins are present in the dust and building materials of WDB [8,9,10,11,12,13,14,15,16]. Therefore, exposure to microbial toxins is most likely underestimated, particularly since mold and bacteria shed fine respirable particulates less than 1 micron in diameter in water-damaged conditions that contain toxins and other by-products [5,6,35,36,37,38,39,40].

The common denominators in these patients included CFS, additional symptoms, a water-damaged environment, indoor mold and urine specimens positive for mycotoxins. OTA was the most common mycotoxin detected in 83% of subjects followed by MT (44%) and AT (13%). Interestingly, more than one of the mycotoxins was also present ranging from 8% (all three mycotoxins) to 23% (MT and OTA) (Table 1). Moreover, the major mycotoxin in the urine of the two families (Table 4, Table 5) was OTA, while MT were positive in two of the subjects. The question that arises is what is the probable role of these mycotoxins in the symptoms experienced by the patients in this study? The Mitochondrial Disease Foundation lists several masquerader health problems that are associated with mitochondrial deficiency, which include the following organs: central nervous system, heart, peripheral nerves, muscles, liver, ears, eyes, pancreas, digestive system and endocrine system. Manifestations of mitochondrial deficiency can include autoimmune disorders, chronic fatigue, neurodegenerative disorders (amyotrophic lateral sclerosis, multiple sclerosis, Parkinson’s disease), depression, other psychiatric disorders, glycogen storage disorders, among others [41]. 

*In vivo* and *in vitro* studies have demonstrated that mycotoxins cause mitochondrial dysfunction. Aflatoxins alter mitochondria as follows: mitochondrial DNA adducts, inhibition of protein synthesis, pleomorphism, disruption of cristae, membrane damage and induction of apoptosis [42,43,44,45]. Trichothecenes have multiple inhibitory effects that include oxidative stress, apoptosis, inhibition of protein, RNA and DNA synthesis, opening of phosphorescent Pt(II)-coporporphyrin (PtCP) and loss of transmembrane potential and mitochondrial translation [46,47,48,49,50]. With respect to OTA the primary thrust has been detecting its role in urinary tract and kidney diseases. However, the research into kidney diseases has shown that OTA is also a mitochondrial poison. Mitochondrial abnormalities resulting from OTA include membrane swelling, disarray of cristae, loss of transmembrane potential, inhibition of succinate cytochrome c reductase and succinate dehydrogenase and inhibition of succinate-supported electron transfer, and activities of the respiratory chain. The toxicity of OTA appears to result from oxidative stress leading to nuclear DNA damage, cytotoxicity and apoptosis [51,52,53,54]. Thus, it appears that mitochondrial dysfunction may be correlated with the presence of CFS and other symptoms in these patients.

The patients presented in this study had multiple symptoms including those consistent with CFS as reported by others [22,23,24,25,26,27,28,29,55,56]. They had increased concentrations of AT, OTA and MT in their urine samples compared to a group of previously published healthy controls. Their health conditions and symptoms in these patients were suggestive of mitochondrial dysfunction as reported in subjects with CFS [32,33,34]. In addition, the symptom complex of these patients was suggestive of mitochondrial disease as reported by the Mitochondrial Disease Foundation [41]. Moreover, AT, OTA and MT can cause mitochondrial damage [42,43,44,45,46,47,48,49,50,51,52,53,54].

## 4. Conclusions

Mycotoxins can be detected in the urine in a very high percentage of patients with CFS. This is in contrast to a prior study of a healthy, non-WDB exposed control population in which no mycotoxins were found at the levels of detection. The majority of the CFS patients had prior exposure to WDB. Environmental testing in a subset of these patients confirmed mold and mycotoxin exposure. We present the hypothesis that mitochondrial dysfunction is a possible cause of the health problems of these patients. The mitochondrial dysfunction may be triggered and accentuated by exposure to mycotoxins.

## References

[1] Klimas N.G., Broderick G., Fletcher M.A. (2012). Biomarkers for chronic fatigue. Brain Behav. Immun..

[2] Komaroff A.L. (2006). Is human herpesvirus-6 a trigger for chronic fatigue syndrome?. J. Clin. Virol..

[3] Asch E.S., Bujak D.I., Weiss M., Peterson M.G.E., Weinstein A. (1994). Lyme disease: An infectious and postinfectious syndrome. J. Rheumatol..

[4] Fikuda K., Strauss S.E., Hickie I., Sharpe M.C., Dobbins J.G., Komaroff A. (1994). The chronic fatigue syndrome: A comprehensive approach to its definition and study. Ann. Intern. Med..

[5] Thrasher J.D., Crawley S. (2009). The biocontaminants and complexity of damp indoor spaces: more than what meets the eyes. Toxicol. Ind. Health.

[6] Täubel M., Sulyok M., Vishwanath V., Bloom E., Turunen M., Järvi K., Kauhanen E., Krska R., Hyvärinen A., Larsson L., Nevalainen A. (2011). Co-occurrence of toxic bacterial and fungal secondary metabolites in moisture-damaged indoor environments. Indoor Air.

[7] Brasel D., Douglas R., Wilson S.C., Straus D.C. (2005). Detection of airborne *Stachybotrys chartarum* macrocyclic trichothecene mycotoxins on particulates smaller than conidia. Appl. Environ. Microbiol..

[8] Gottschalk C., Bauer J., Meyer K. (2008). Detection of Satratoxins G and H in indoor air from a water-damaged building. Mycopathologia.

[9] Smoragiewicz W., Cossette B., Boutard A., Krzystyniak K. (1993). Trichothecene mycotoxins in the dust of ventilation systems of office buildings. Int. Arch. Occup. Environ. Health.

[10] Polizzi V., Delmulle B., Adams A., Moretti A., Susca A., Picco A.M., Rosseel Y., Kindt R., Van Bocxlaer J., De Kimpe N., Van Peteghem C., De Saeger S. (2009). JEM spotlight: fungi, mycotoxins and microbial volatile organic compounds in mouldy interiors of water-damaged buildings. J. Environ. Monit..

[11] Bloom E., Nyman E., Must A., Pehrson C., Larsson L. (2009). Molds and mycotoxins in indoor environments - a survey in water-damaged buildings. J. Occup. Environ. Hyg..

[12] Tuomi T., Reijula K., Johnsson T., Hemminki K., Hintikka E.L., Lindroos O., Kalso S., Koukila-Kähkölä P., Mussalo-Rauhamaa H., Haahtela T. (2000). Mycotoxins in crude building materials from water-damaged buildings. Appl. Environ. Microbiol..

[13] Engelhart S., Loock A., Skutlarek D., Sagunski H., Lommel A., Färber H., Exner M. (2002). Occurrence of toxigenic *Aspergillus versicolor* isolates and sterigmatocystin in carpet dust from damp indoor environments. Appl. Environ. Microbiol..

[14] Peitzsch M., Sulyok M., Täubel M., Vishwanath V., Krop E., Borràs-Santos A., Hyvärinen A., Nevalainen A., Krska R., Larsson L. (2012). Microbial secondary metabolites in school buildings inspected for moisture damage in Finland, the Netherlands and Spain. J. Environ. Monit..

[15] Polizzi V., Adams A., Malysheva S.V., De Saeger S., Van Peteghem C., Moretti A., Picco A.M., De Kimpe N. (2012). Identification of volatile markers for indoor fungal growth and chemotaxonomic classification of *Aspergillus* species. Fungal Biol..

[16] Polizzi V., Adams A., Picco A.M., Adriaens E., Lenoir J., Van Peteghem C., De Saeger S., De Kimpe N. (2011). Influence of environmental conditions on production of volatiles by *Trichoderma atroviride* in relation with sick building syndrome. Build. Environ..

[17] Leema G., Kaliamurthy J., Geraldine P., Thomas P.A. (2010). Keratitis due to *Aspergillus flavus*: Clinical profile, molecular identification of fungal strains and detection of aflatoxin production. Mol. Vis..

[18] Matsumura M., Mori T. (1998). Detection of aflatoxins in autopsied materials from a patient infected with *Aspergillus flavus*. Jpn. J. Med. Mycol..

[19] Lewis R.E., Wiederhold N.P., Lionakis M.S., Prince R.A., Kontoyiannis D.P. (2005). Frequency and species distribution of gliotoxin-producing *Aspergillus* isolates recovered from patients at a tertiary-care cancer center. J. Clin. Microbiol..

[20] Lewis R.E., Wiederhold N.P., Chi J., Han X.Y., Komanduri K.V., Kontoyiannis D.P., Prince R.A. (2005). Detection of gliotoxin in experimental and human aspergillosis. Infect. Immun..

[21] Hooper D.G., Bolton V.E., Guilford F.T., Straus D.C. (2009). Mycotoxin detection in human samples from patients exposed to environmental molds. Int. J. Mol. Sci..

[22] Thrasher J.D., Gray M.R., Kilburn K.H., Dennis D., Yu A. (2012). A water-damaged home and health of occupants: A case study. J. Environ. Public Health.

[23] Lieberman S.M., Jacobs J.B., Lebowitz R.A., Fitzgerald M.B., Crawford J., Feigenbaum B.A. (2011). Measurement of mycotoxins in patients with chronic rhinosinusitis. Otolaryg. Head Neck Surg..

[24] Rea W.J., Didriksen N., Simon T.R., Pan Y., Fenyves E.J., Griffiths G. (2003). Effects of toxic exposure to molds and mycotoxins in building-related illnesses. Arch. Environ. Heath.

[25] Rea W., Pan Y., Griffiths B. (2009). The treatment of patients with mycotoxin-induced disease. Toxicol. Indust. Health.

[26] Curtis L., Leiberman A., Stark M., Rea W., Vetter M. (2004). Adverse Health Effects of Indoor Molds. J. Aust. Coll. Nutr. Env. Med..

[27] Campbell A., Thrasher J.D., Gray M.R., Vojdani A. (2004). Mold and mycotoxins: Effects on the neurological and immune systems in humans. Adv. Appl. Microbiol..

[28] Chester A.C., Levine P. (1994). Concurrent sick building syndrome and chronic fatigue syndrome: Epidemic neuromyasthenia revisited. Clin. Infect. Dis..

[29] Gray M.R., Thrasher J.D., Crago R., Madison R.A., Arnold L., Campbell A.W., Vojdani A. (2003). Mixed mold mycotoxicosis: Immunological changes in humans following exposure to water-damaged buildings. Arch. Environ. Health.

[30] Edmondson D.A., Nordness M.E., Zacharison M.C., Kurup V.P., Fink J.N. (2005). Allergy and “toxic mold syndrome.”. Ann. Allergy Asthma Immunol..

[31] Vesper S., McKinstry C., Cox D., Dewalt G. (2009). Correlation between ERMI values and other moisture and mold assessments of homes in the American healthy home survey. J. Urban. Health.

[32] Wong R., Lopaschuk G., Zhu G., Walker D., Catellier D., Burton D., Teo K., Collins-Nakai R., Montague T. (1992). Skeletal muscle metabolism in the chronic fatigue syndrome. In vivo assessment by 31P nuclear magnetic resonance spectroscopy. Chest.

[33] Lane R.J., Barrett M., Taylor D., Kemp G.J., Lodi R. (1998). Heterogeneity in chronic fatigue syndrome: evidence from magnetic resonance spectroscopy of muscle. Neuromuscul. Disord..

[34] Booth N.E., Myhill S., Mclaren-Howard J. (2012). Mitochondrial dysfunction and the pathophysiology of Myalgic Encephalomyelitis/Chronic Fatigue Syndrome (ME/CFS). Int. J. Clin. Exp. Med..

[35] Gorny R.L. (2004). Filamentous microorganisms and their fragments in indoor air-A review. Ann. Agric. Environ. Med..

[36] Cho S.-H., Seo S.-C., Schmechel D., Grinshpun A.G., Reponen T. (2005). Aerodynamic characteristics and respiratory deposition of fungal fragments. Atmos. Environ..

[37] Reponen T., Seo S.-C., Grimsley F., Lee T., Crawford C., Grinshpun S.A. (2007). Fungal fragments in moldy houses: A field study in homes in New Orleans and Southern Ohio. Atmos. Environ..

[38] Ammann H.M. (2005). Mycotoxins in indoor environments. Mycotoxin Res..

[39] Straus D.C. (2009). Molds, mycotoxins and sick building syndrome. Toxicol. Indust. Health.

[40] Straus D.C. (2011). The possible role of fungal contaminants in sick building syndrome. Front. Biosci..

[41] Mitochondrial Disease Foundation Mitochondrial Disease in Adults. http://www.umdf.org/site/pp.aspx?c=8qKOJ0MvF7LUG&b=7934631.

[42] Bhat N.K., Emeh J.K., Niranjan B.G., Avadhani N.G. (1982). Inhibition of mitochondrial protein synthesis during early stages of aflatoxin B_1_ carcinogenesis. Cancer Res..

[43] Shamsuddin A.M., Harris C.C., Hinzman M.J. (1987). Localization of aflatoxin B2-nucleic acid adducts in mitochondria and nuclei. Carcinogenesis.

[44] Rainbow L., Maxwell S.M., Hendrickes R.G. (1994). Ultrastructural changes in murine lymphocytes induced by aflatoxin B1. Mycopathologia.

[45] Yang X.J., Lu H.Y., Li Z.Y., Bian Q., Qiu L.L., Li Z., Liu Q., Li J., Wang X., Wang S.L. (2012). Cytochrome P450 2A13 mediates aflatoxin B1-induced cytotoxicity and apoptosis in human bronchial epithelial cells. Toxicology.

[46] Rocha A., Ansari K., Doohan F.M. (2005). Effects of trichothecene mycotoxins on eurkaryotic cells: a review. Food Addit. Contam..

[47] Chaudhari M., Jayaraj R., Bhaskar A.S., Lakshmana P.V. (2009). Oxidative stress induction by T-2 toxin causes DNA damage and triggers apoptosis via caspase pathway in human cervical cancer cells. Toxicology.

[48] Bin-Ulmer M.A., McLaughlin J.E., Basu D., McCormick S., Turner N.E. (2011). Trichothecene mycotoxins inhibit mitochondrial translation-implication for the mechanism of toxicity. Toxins (Basel).

[49] Bouaziz C., Sharif el dein O., El Golli E., Abid-Essefi S., Brenner C., Lermaire C., Bacha H. (2006). Different apoptic pathways induced by zeralenone, T-2 toxin and ochratoxin A in human hepatoma cells. Toxicology.

[50] Bouaziz C., Martel C., Sharaf el dein O., Abid-Essefi S., Brenner C., Lemaire C., Bacha H. (2009). Fusarial toxin-induced toxicity in culture cells and in isolated mitochondria involve PTPC-dependent activation of the mitochondrial pathway of apoptosis. Toxicology.

[51] Kamp H.G., Eisenbrand G., Schlatter J., Worth K., Janzowski C. (2005). Ochratoxin, A: Induction of oxidative DNA damage, cytotoxicity and apoptosis in mammalian cell lines and primary cells. Toxicology.

[52] Wei Y.H., Lu C.Y., Lin T.N., Wei R.D. (1985). Effect of ochratoxin A on rat liver mitochondrial respiration and oxidative phosphorylation. Toxicology.

[53] Prabu P.C., Dwivedi P., Sharma A.K. (2013). Toxicopathological studies on the effects of aflatoxin B(1), ochratoxin A, and the interaction in New Zealand white rabbits. Exper. Toxicol. Pathol..

[54] Arbillaga L., Azqueta A., Ezpeleta O., López de Cerain A. (2006). Oxidative damage induced by ochratoxin A in HK-2 human kidney cells: evidence of the relationship with cytotoxicity. Mutagenesis.

[55] Kilburn K.H. (2009). Neurobehavioral and pulmonary impairment in 105 adults with indoor exposure to indoor molds compared to 100 exposed to chemicals. Toxicol. Indust. Health.

[56] Empting L.D. (2009). Neurologic and neuropsychiatric syndrome features of mold and mycotoxin exposure. Toxicol. Indust. Health.

